# A new genus of spider beetle (Coleoptera, Ptinidae) from western Peru

**DOI:** 10.3897/zookeys.934.38670

**Published:** 2020-05-19

**Authors:** T. Keith Philips, Kyle A. Whorrall, Olivia M. Gearner, Jean-Bernard Huchet

**Affiliations:** 1 Systematics and Evolution Laboratory, Department of Biology, Western Kentucky University, 1906 College Heights Blvd., Bowling Green, KY 42101-3576, USA Western Kentucky University Bowling Green United States of America; 2 Muséum National d’Histoire Naturelle, UMR 7205 ISYEB, Institut de Systématique, Evolution et Biodiversité, 45, rue Buffon, F-75005 Paris, France Muséum National d’Histoire Naturelle Paris France

**Keywords:** Atacama Desert, Bostrichoidea, diversity

## Abstract

A new genus of flightless spider beetle from Peru with two new species is described. It is characterized by unique heart-shaped fused elytra and a broad pronotum with five basal depressions. The characters of this new genus and species are illustrated and discussed and the possible phylogenetic placement of this taxon is also included.

## Introduction

The spider beetle fauna of South America currently includes ca. 100 described species placed in 11 genera. Little recent work has been done on the group with the exception of publications by Bellés on *Ptinus* Linnaeus (1984, 1986), *Bellesus* Özdikmen (as *Arachnomimus* Bellés 1985 and see Özdikmen 2010), *Prosternoptinus* Bellés (1985), and *Tropicoptinus*Bellés (1998), by Borowski on *Trigonogenius* Solier (2000, 2006) and earlier studies on the myrmecophilous fauna (*Gnostus* Westwood and *Fabrasia* Martinez & Viana) by Lawrence and Reichardt (1966). All other descriptive work occurred before 1939 and mainly by Maurice Pic on *Ptinus* (e.g., Pic 1900, 1936, 1939). The fauna is still largely undocumented and possibly most morphologically diverse in the more xeric regions of this continent, a habitat that often leads to highly modified external morphologies and flightlessness (Philips 2000). In particular, additional hidden diversity exemplified by this new genus may be discovered in the drier areas of the west from southern Ecuador through Peru to Chile and east into Argentina. Part of this region in the southern coastal portion is the hyper-arid Atacama Desert.

The two new species that make up this new genus are known only from Peru. Surprisingly one of the new species discovered was in material from the Ditsong Museum located in Pretoria, South Africa. The second species was collected by one of the authors (JBH) during archaeoentomological investigations on the emblematic site of Huaca de la Luna in 2009. Documenting this genus and concomitant new species increases the known morphological range within spider beetles and will make the name available for unpublished phylogenetic studies.

## Taxonomy

### Cordielytrum

Taxon classificationAnimaliaColeopteraPtinidae

Philips gen. nov.

2BBE66B5-584A-504B-97B6-D7919CAB3803

http://zoobank.org/2F6CB8B5-F409-4C16-9FB0-BD958A68CB5F

**Figure 1.**
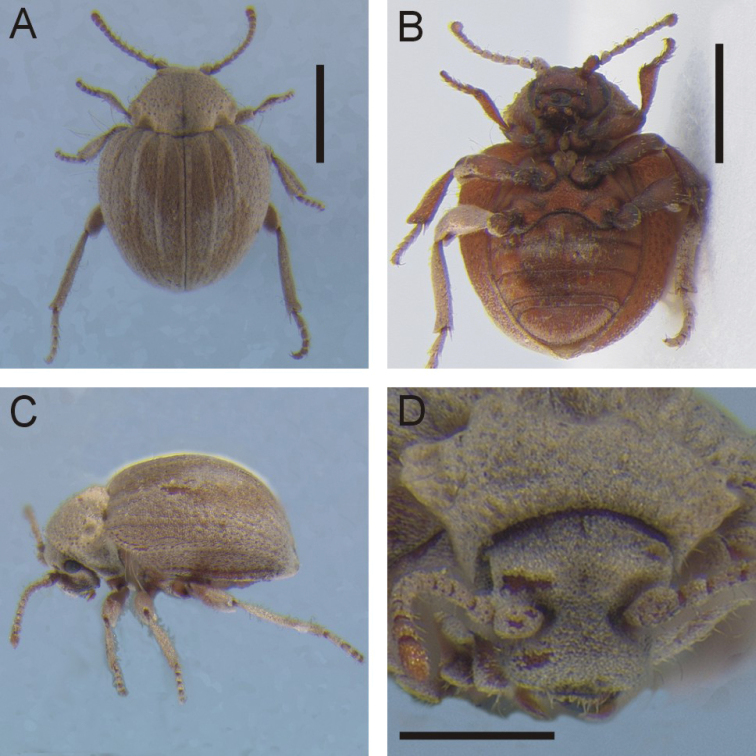
*Cordielytrum peruvianum* sp. nov. **A** Dorsal habitus **B** ventral habitus **C** lateral view **D** frontal view. Scale bars: 1.0 mm (**A, B**), 0.5 mm (**D**).

**Figure 2.**
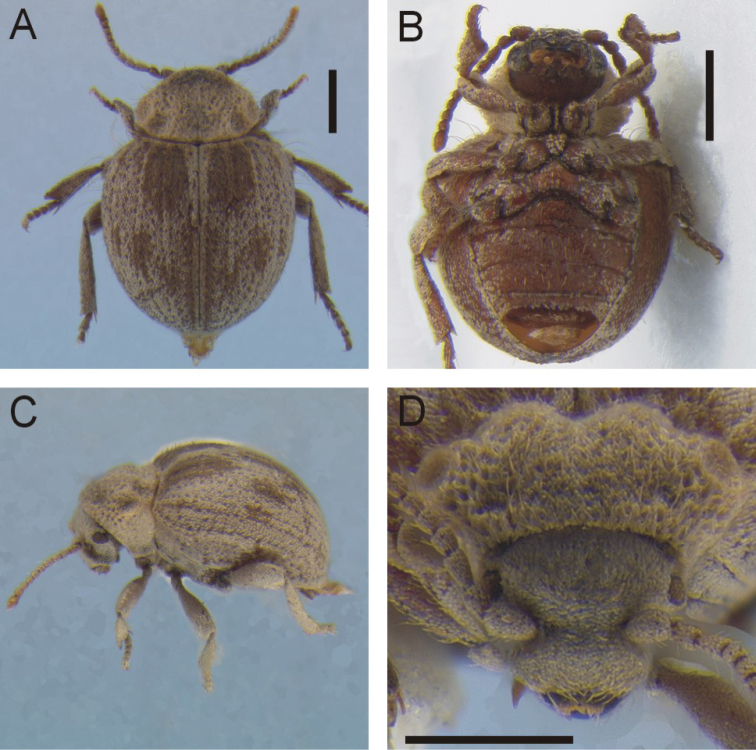
*Cordielytrum pulchrum* sp. nov. **A** Dorsal habitus **B** ventral habitus **C** lateral view **D** frontal view. Scale bars: 1.0 mm (**A, B**), 0.5 mm (**D**).

**Figure 3.**
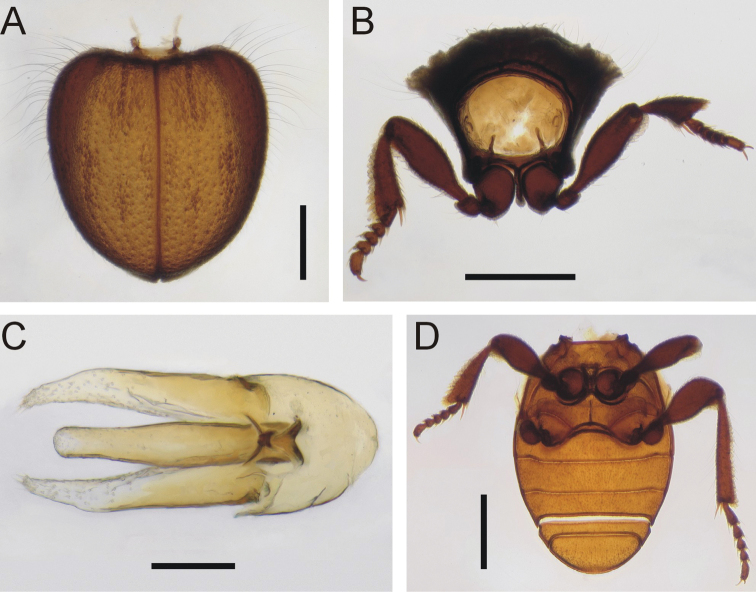
*Cordielytrum pulchrum* sp. nov. **A** Elytra, dorsal view **B** prothorax, frontal view, **C** aedeagus, dorsal view **D** meso- and metaventrites and abdominal ventrites. Scale bars: 0.5 mm (**A, B, D**), 0.1 mm (**C**).

**Figure 4.**
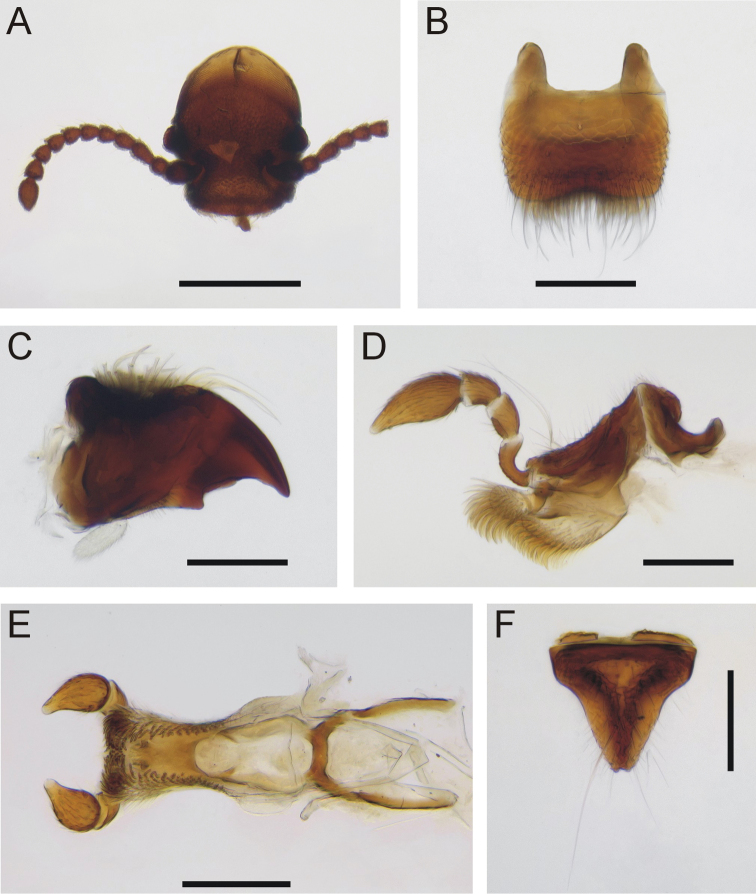
*Cordielytrum pulchrum* sp. nov. **A** Head, frontal view **B** labrum, dorsal view **C** mandible, ventral view **D** maxilla, ventral view **E** labium, dorsal view **F** mentum, ventral view. Scale bars: 0.5 mm (**A**), 0.1 mm (**B–F**).

Figures 1 , 2 , 3 , 4

#### Type species.

*Cordielytrum peruvianum* Whorrall & Philips.

#### Diagnosis.

This genus can be recognized by the heart-shaped pair of fused elytra and the dense appressed setal scale covering (Figs 1, 2). There are also very elongate erect setae on the lateral edge of the pronotum and humeral area of the elytra that extend laterally and curve slightly posteriorly apically. Near the pronotal base are five depressions; one large median and two smaller ones positioned more laterally on each side. There is also a distinct and relatively large pocket on the head positioned below the eye and extending to the lateral edge of the clypeus. Currently the two species in this genus are known only from Peru.

#### Description.

***Body***: small, length approximately 2 to 2.5 mm, ovoid, convex but slightly flattened dorso-ventrally, dorsally body surface completely obscured with appressed or recumbent setae, scale-like especially on head and elytra.

***Head*** (Fig. 4A): Eyes not visible dorsally, small, ovoid, slightly rounded ommatidial surface facing slightly upwards; vertex very slightly convex, antennal fossae generally indistinct, smoothly rounded edges, but more distinct dorsally and laterally absent; a large distinct pocket extending laterally from fossa through to gena between eye and lateral edge of clypeus; antennae short in length, no longer than the maximum width of the pronotum, 11 antennomeres, 4–10 relatively stout, only slightly longer than wide, second antennomere inserted off center of scape near lateral edge; interantennal space between antennal insertions wide, width approximately the same as length of scape, flat with no carina between antennal fossae; clypeus triangular, maximal width ≈ 4/5 width of head measured adjacent to pronotum, labrum (Fig. 4B) ca. 1/3 width of clypeus, anterior edge very slightly emarginated in middle; mandible apex acutely pointed, medial tooth present (Fig. 4C); maxillary and labial palps with four and three palpomeres respectively (Fig. 4D, E); mentum triangular, with small triangular cavity at middle (Fig. 4F).

***Pronotum*** (Fig. 3B): Strongly transverse with five depressions adjacent to the posterior margin, one large median and two smaller laterally on each side (Figs 1A, 2A); elongate erect setae on lateral edge extending laterally.

***Elytra*** (Fig. 3A): Heart-shaped, convex, fused along suture; longitudinal carinae at least at base and sometimes visible the entire length of each elytron, short suberect setae on carinae; relatively elongate setae on humeral area that extend laterally and curve posteriorly near their apex; 2–3 irregular fine puncture rows between each carina.

***Thorax*** (Fig. 3D): Broader than long; prosternal process narrow anteriorly, widening posteriorly, teardrop- shaped, extending posteriorly to same distance as procoxae do posteriorly, extending slightly into mesoventrite; visible part of mesoventrite heart-shaped with a truncate tip posteriorly, between coxae slightly longer than wide, slightly shorter than length of metaventrite at middle; posterior margin of metaventrite broadly emarginated; mesoventral-mesepisternal and metaventral-metepisternal sutures both visible.

***Abdominal ventrites*** (Fig. 3D): Ventrites broad, ca. 2/3 total width of body measured at/opposite ventrite base, all sutures visible, first three ventrites connate, length at middle compared to laterally slightly shorter except fifth where longest at middle; first and second approximately equal in length, third slightly shorter, fourth distinctly shortest, fifth distinctly longest.

***Legs*** (Fig. 3B, D): moderate in length, femora widest near middle; tibia gradually expanded toward apex, pro- and mesotibiae similar in length to their respective femora, metatibiae distinctly longer; tarsomeres 2–4 ca. as wide as long, 1^st^ and 5^th^ ca. equal in length; procoxae and mesocoxae approximately rounded, procoxae slightly smaller in diameter than mesocoxae, metacoxae transverse, fused with metaventrite.

***Male genitalia*** (Fig. 3C): relatively simple; parameres and median lobe relatively stout, parameres lacking setal clumps or other modifications.

#### Etymology.

The generic name is derived from *cordi* = Latin for heart and *elytrum* = Greek for sheath in reference to the fused elytra that figuratively resemble an ideographic image of a heart.

#### Remarks.

Sexual dimorphism externally is not apparent.

#### Distribution.

**Figure 5.**
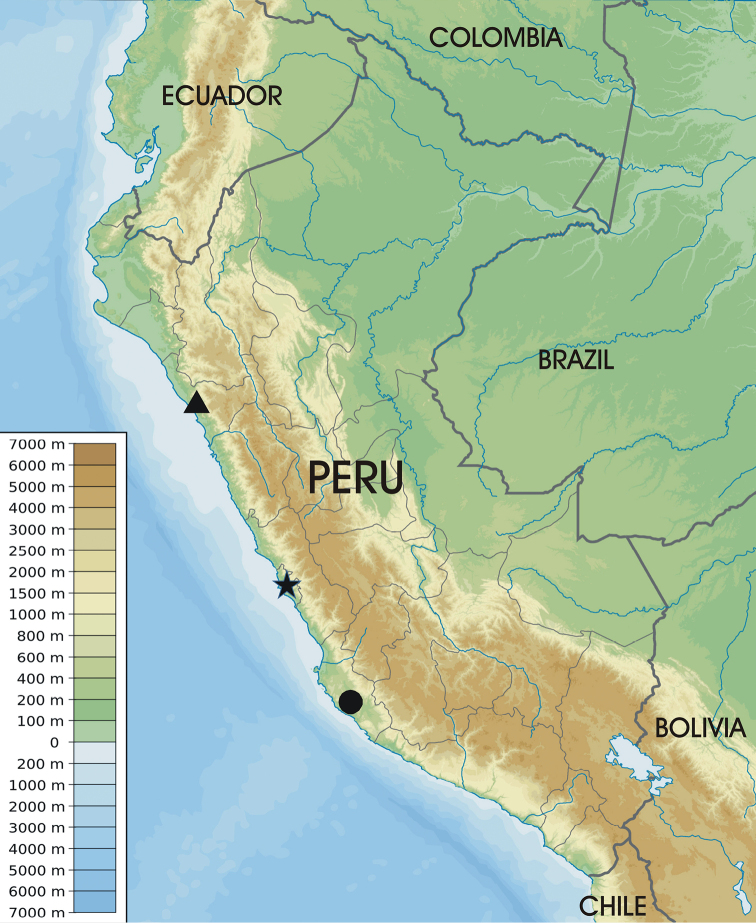
Distribution of *Cordielytrum peruvianum* (circle) and *C. pulchrum* (triangle). The position of the city of Lima is also indicated (star).

Members of this genus appear to be denizens of xeric coastal areas in Peru (Fig. 5). Based on the locations of the two known species, there is a separation of over 700 km. Recent fieldwork in Peru has resulted in the collection of additional undescribed species in the south with one ca. 730 km straight line distance from Lima at ca. 17° latitude (Whorrall and Philips, unpublished). The discovery of even more undocumented species with further sampling should be expected.

#### Ecology.

Currently no information on the ecology is known with the exception of the northernmost species that was collected via traps baited with a local corn beer known as chicha: this fluid may have been attractive as a food and/or moisture source. Based on recent collections and current rearing experiments (Philips and Whorrall, unpublished), larvae feed on cat dung and likely any other type in their vicinity, such as that from other mammals, birds, or lizards.

### Cordielytrum peruvianum

Taxon classificationAnimaliaColeopteraPtinidae

Whorrall & Philips sp. nov.

26D291FA-FBDE-56EF-8C36-5609C0F9A1BB

http://zoobank.org/551A269D-CDA6-4328-B04F-9BE278A60B8D

Figures 1 , 5

#### Type material.

***Holotype.* Peru**: Ica, coastal dunes, Seely, 15.I.1980. Deposited in the Museo de Historia Natural, Lima Peru. ***Paratypes*** (6): Same data as holotype. Paratypes have been deposited in the Ditsong Museum and the collections of the authors (TKPC, JBHC, KAWC).

#### Diagnosis.

This species is distinguished from its congener by its more rotund shape and the narrow light tan scales covering the elytra. It is known only from the type locality in the Ica Region of Southern Peru.

#### Description.

Body small, compact, subovate, convex; head, pronotum, and elytra tan colored. Length (anterior of pronotum to apex of elytra) 2.16 – 2.76 (μ = 2.55 ± 0.20) mm (*N* = 7).

***Head***: densely covered in light tan, depressed, ovoid scales completely covering surface, less dense laterally, with pronounced superantennal carinae; antennomeres 1–9 densely squamous, ultimate and penultimate antennomeres with simple setae, not obscuring surface, antennomeres 1–3 and 11 slightly longer than wide, others subequal.

***Pronotum***: setose with scales anteriorly and longer densely matted setae posteriorly; short, erect setae sparsely placed throughout, longer at posterio-lateral edge, arising from cavities formed within matted setae, cavities distinctly larger laterally; medial cavity deep, when viewed from above, extending nearly half total length but border absent at middle, extending as a shallow groove anteriorly; two posterio-lateral cavities on each side, deep and distinct.

***Elytra***: surface densely covered by narrow scales, giving a finely rugose appearance; lateral edge at anterior ¼ with row of sparsely placed bristly very long setae; four prominent longitudinal carinae extending length of each elytron, including one at suture; indistinct very shallow depressions in ~ two rows between carinae; background cuticle color light reddish brown.

***Ventral surface***: Pro- and mesoventrites with matted setation similar to pronotum; metaventrite and abdominal ventrites with scales similar to elytra. Femora increasing in width from base to apex, girth reaching maximum around midpoint; tibiae increasing in width from base to apex, girth increasing throughout basal third then remaining equal thereafter; tarsomere 1 ca. twice as long as 2–4, ⅓ longer than 5, 2–4 sub-equal in length.

#### Etymology.

The specific name *peruvianum* refers to the country in South America where this species was discovered.

#### Remarks.

This species is from the Ica Region, Peru (Fig. 5). The precise location is unclear as the only additional information on the label is “coastal dunes.”

### Cordielytrum pulchrum

Taxon classificationAnimaliaColeopteraPtinidae

Whorrall & Philips sp. nov.

80C6E77C-543B-53FB-A0B8-8E4BCEEEA813

http://zoobank.org/DA2130D6-0A9C-4230-BD14-FB2381F2AB3E

Figures 2 , 5

#### Type material.

***Holotype.*** Peru: Trujillo, Huaca de la Luna, Plateforme Uhle, J. B. Huchet lgt.; Piège à “Chicha, (bière de maïs), (J6)13/05/2009). Holotype deposited in the Muséum national d’Histoire naturelle, Paris, France. ***Paratypes*** (18), same data as the holotype (13); Peru- Trujillo, Huaca de la Luna, 6. V–1.VII. 2009, J.B Huchet / A. Chauchat (5). Paratypes have been deposited in the Muséum national d’Histoire naturelle, Museo de Historia Natural, Lima, and the collections of the authors (TKPC, JBHC, KAWC).

#### Diagnosis.

This species is distinguished from *C. peruvianum* by its slightly more elongate shape, the vestiture of broad, ovate tan and dark brown scales on its elytra. Currently this species is only known from the type locality in Northern Peru.

#### Description.

Body small, compact, subovate, convex; head and pronotum tan, elytra mottled tan and dark brown. Length (anterior of pronotum to posterior of elytra) 1.84– 2.44 (μ = 2.13 ± 0.22) mm (*N* = 15).

***Head*** densely covered in light tan, depressed, ovoid scales completely covering surface, less dense laterally, with subtle superantennal carina; antennomeres 1–9 densely squamous, ultimate and penultimate antennomeres with simple setae; antennomeres 1–3 and 11 ca. twice as long as wide, others subequal.

***Pronotum*** setose with scales anteriorly and longer densely matted setae posteriorly; short, erect setae sparsely placed throughout, longer at posterio-lateral edge, arising from cavities formed within matted setae, cavities distinctly larger laterally; medial cavity with poorly defined border, moderate in depth, when viewed from above, extending nearly one third of total length; two posterio-lateral cavities on each side, somewhat more distinct than medial cavity.

***Elytra*** surface densely covered by broad, ovate scales, giving a coarsely rugose appearance; lateral edge at anterior ⅓ of each elytron with row of long densely placed bristly very long setae; four low longitudinal carinae extending length of each elytron, including one at suture; deep depressions in ~2 rows between carinae; background cuticle color dark reddish brown.

***Ventral surface***: Pro-, meso-, and metaventrites and abdominal ventrites with scales similar to elytra. Femora increasing in width from base to apex, girth reaching maximum around midpoint; tibiae increasing in width from base to apex, girth increasing throughout basal third then remaining equal thereafter; tarsomere 1 ca. twice as long as 2–4, ⅓ longer than 5, 2–4 sub-equal in length.

**Etymology.** The name derives from the attractive variegated pattern of dark and light-colored setae on the elytral surface.

**Figure 6.**
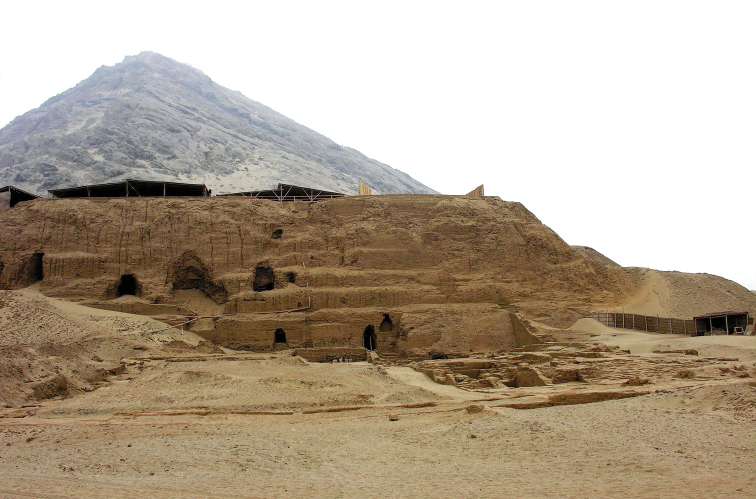
The archaeological site of Huacas de Moche, located along the pacific coastal desert, in the vicinity of Trujillo, 550 km north of Lima, Peru. This is the type locality of *Cordielytrum pulchrum* sp. nov.

**Remarks.** Many well-preserved, partial remains of this new species were initially recovered within organic material from pre-Columbian Mochica graves at the emblematic archaeological site of Huacas de Moche, located along the pacific coastal desert, in the vicinity of Trujillo, 550 km north of Lima, Peru. This archaeological complex includes two monumental pyramids built as a series of platforms: Huaca del Sol (Temple of the Sun) and Huaca de la Luna (Temple of the Moon), separated by a vast urban centre (Chauchat et al. 2009) (Fig. 6). The archaeological remains were directly associated with human skeletons or from inside ceramic vessels placed as offerings. In order to collect specimens of this “subfossil” species, pitfall traps were placed on site by one of the authors (JBH) baited with meat, rotten fruits or with a local corn beer called *chicha.* This fluid may have been attractive as a food and/or moisture source since this permitted collection of all specimens of the type-series.

## Discussion

The most plausible sister lineage is the genus *Trigonogenius*, as both are somewhat comparable morphologically and are found in the same region of South America. To support this hypothesis, an attempt to acquire a sample of DNA from a dried specimen was done but amplification attempts failed. Hence more complete data to support relationships and to truly understand where the ancestry of this taxon lies will have to wait until fresh material is processed and sequenced.

Further collecting efforts in coastal Peru south of Lima has resulted in the discovery of additional undescribed species of this genus. How many more species remain to be found in this poorly surveyed part of South America remains unknown.

## Supplementary Material

XML Treatment for Cordielytrum

XML Treatment for Cordielytrum peruvianum

XML Treatment for Cordielytrum pulchrum
